# Cerebellar rTMS in PSP: a Double-Blind Sham-Controlled Study Using Mobile Health Technology

**DOI:** 10.1007/s12311-021-01239-6

**Published:** 2021-02-05

**Authors:** Andrea Pilotto, Maria Cristina Rizzetti, Alberto Lombardi, Clint Hansen, Michele Biggi, Giacomo Verzeroli, Antonella Martinelli, Robbin Romijnders, Barbara Borroni, Walter Maetzler, Alessandro Padovani

**Affiliations:** 1 Neurology Unit, Department of Clinical and Experimental Sciences, University of Brescia, P.zale Spedali Civili, 1, 25123 Brescia, Italy; 2 Parkinson’s Disease Rehabilitation Unit, FERB Onlus Trescore Balneario, Bergamo, Italy; 3 Department of Neurology, Christian-Albrechts-University of Kiel, Kiel, Germany; 4 Physical Therapy Unit, Milano Bicocca University, Bergamo, Italy

**Keywords:** Progressive supranuclear palsy, Repetitive transcranial magnetic stimulation, Mobile health technology, Postural instability

## Abstract

There are no effective treatments in progressive supranuclear palsy (PSP). The aim of this study was to test the efficacy of theta burst repetitive transcranial magnetic stimulation (rTMS) on postural instability in PSP. Twenty PSP patients underwent a session of sham or real cerebellar rTMS in a crossover design. Before and after stimulation, static balance was evaluated with instrumented (lower back accelerometer, Rehagait®, Hasomed, Germany) 30-s trials in semitandem and tandem positions. In tandem and semitandem tasks, active stimulation was associated with increase in time without falls (both *p*=0.04). In the same tasks, device-extracted parameters revealed significant improvement in area (*p*=0.007), velocity (*p*=0.005), acceleration and jerkiness of sway (*p*=0.008) in real versus sham stimulation. Cerebellar rTMS showed a significant effect on stability in PSP patients, when assessed with mobile digital technology, in a double-blind design. These results should motivate larger and longer trials using non-invasive brain stimulation for PSP patients.

**Supplementary Information:**

The online version contains supplementary material available at 10.1007/s12311-021-01239-6.

## Introduction

Progressive supranuclear palsy (PSP) is a neurodegenerative disorder characterised by akinetic rigid syndrome with ocular motor dysfunction, early postural instability and falls [1].

Despite potential limited benefit from dopaminergic drugs, there are still no effective treatments available for postural instability and falls. Recent imaging and neuropathology studies revealed a reduced volume of the cerebellum with Tau accumulation in PSP patients [2, 3]. These evidences suggest that cerebellum may be a potential target for non-invasive stimulation, as already recently demonstrated for multiple sclerosis [4].

Accordingly, neurophysiological studies demonstrated an impairment in functional connectivity between the cerebellar hemispheres and contralateral primary motor cortex (cerebellar brain inhibition, CBI) [5, 6]. A preliminary, open-label trial with 10 PSP patients showed an improvement of CBI using theta burst repetitive cerebellar transcranial magnetic stimulation (rTMS) [7]. Moreover, a case study showed improvement of posturography parameters secondary to cerebellar stimulation in two PSP patients [8].

Based on these promising results, we aimed at evaluating the effect of a single-session cerebellar rTMS in PSP patients. We applied a double-blind sham-controlled crossover design, including a standardised assessment of static balance using mobile health technology.

## Methods

### Study Cohort

Consecutive PSP patients were recruited and underwent a review of the medical history, a neurological examination including the PSP rating scale (PSPRS) [9] and a comprehensive cognitive and behavioural assessment [10]. Inclusion criteria were (1) clinical diagnosis of probable PSP according to current criteria [1], (2) the ability to stand alone without support and (3) the ability to walk at least 3 m without aid. Exclusion criteria were (1) dementia, (2) vestibular/proprioceptive or sensory abnormalities and (3) any contraindication to perform brain stimulation.

All subjects gave written informed consent prior to participation. The local ethics committee approved the study (protocol 193/16), recorded as NCT04222218 in clinicaltrial.gov. The study was performed in accordance with the Declaration of Helsinki.

### Repetitive Transcranial Magnetic Stimulation

Each patient received both rTMS and sham cerebellar single-session stimulations in randomised order in two different sessions performed at the same time of the day, separated by at least 2 weeks. The patient and the examiner were blind to the type of rTMS delivered, applied by another experimenter. Repetitive cerebellar theta burst stimulation was performed by Duo-Mag XT100 (Deymed -Horonov, Czech Republic) according to the protocol described by Brusa and coauthors [7]. The coil was placed tangentially to the skull over the lateral cerebellum 1 cm inferior and 3 cm right to the inion. Three 50-Hz pulses were repeated at a rate of 5 Hz; 20 trains of 10 bursts with 8-s intervals for a total of 600 pulses and for total time of 240 seconds were applied [7]. The intensity of rTMS was set at the 80% of the resting motor threshold obtained in the left motor cortex for each subject. For sham simulation, a spacer was attached to the coil; the stimulation parameters, the coil position and the sound were identical to the active condition.

### Dynamic Mobility and Mobile Health Technology-Instrumented Static Balance Assessment

All subjects underwent a clinical evaluation including the Tinetti test, the Short Physical Performance Battery (SPPB), the Timed Up and Go test and the Functional Reach test (FR) before and after stimulation [11]. Static balance was tested before and after each stimulation with four tasks of 30-s duration, respectively: tandem and semitandem stance with eyes closed and with eyes open (Supplementary Figure 1). Primary endpoint was changes in the time in this position without support. For mobile health technology secondary outcomes, an inertial sensor unit (IMU) with 100 Hz sampling frequency (Rehagait®, Hasomed GmbH, Magdeburg, Germany) was fixed at the level of the third lumbar spine segment close to the centre of mass [11]. Acceleration signals were processed and calculated as previously described [12]. The following sway parameters were extracted: area, mean velocity, mean acceleration (root mean square - RMS), jerk (indicating smoothness of compensatory movements) and mean frequency [13]. Mean velocity, RMS and jerk were calculated for both anteroposterior (AP) and medio-lateral (ML) directions.

### Statistical Analyses

Differences in baseline performances between real and sham trials were evaluated using Mann-Whitney test. A two-way repeated-measures analysis of variance (ANOVA) was run to determine the effect of the different treatments over time on assessment, adjusted for baseline values and the sequence of stimulation (real-sham vs sham-real). Significance was set to *p*=0.05, and SPSS software (version 21; SPSS, Inc., Chicago, IL) was used.

## Results

### Study Cohort and rTMS

Twenty PSP patients entered the study (mean age 74 + 4 years, 13 males and 7 females). The mean disease duration was 3.8 + 1.2 years, and the mean score on the PSP rating scale was 29 + 5 points, with a mean levodopa equivalent dose of 417 + 89 mg/day. All patients presented with postural instability, as reflected by specific PSPRS items and Tinetti scale. The rTMS protocol was well tolerated by all participants; side effects were neither reported nor observed during and after the stimulations.

There was no statistically significant association between type of stimulation and perception of patients (*p*=0.89, Fisher’s exact test), suggesting that real rTMS could not be distinguished from sham stimulation.

### Dynamic Mobility and Mobile Health Technology-Instrumented Static Balance Assessment

**Fig. 1:**
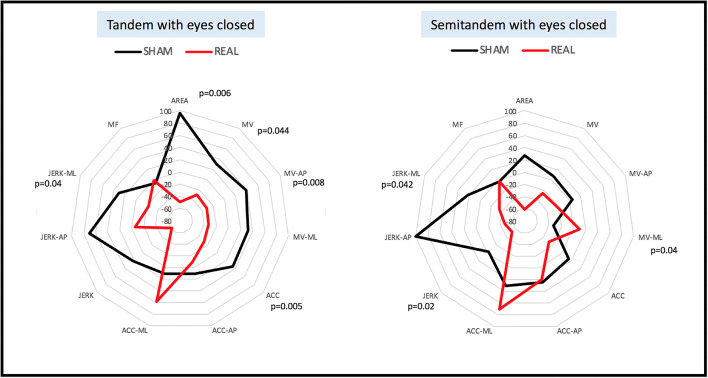
Spider graphs showing the changes (in percentage) of static balance parameters from tandem stance and semitandem stance with eyes closed in patients with PSP, compared to values obtained before the sham versus real intervention. Significant differences are presented with *p*-values. ACC, acceleration; AP, anterior-posterior; MF, mean frequency; ML, medio-lateral; MV, mean velocity

All patients were able to complete the 30-s semitandem/tandem stance trials with eyes open, respectively. Sixteen participants completed the semitandem trial with eyes closed, and 14 the tandem trial with eyes closed. No differences in baseline performances in instrumented tests were detected for each task between real and sham stimulation. In both eyes closed conditions, the participants were able to stay longer without support after the real rTMS, compared to sham stimulation. Moreover, in the tandem stance with eyes closed condition, the real intervention showed an improvement of the following parameters, compared to sham intervention: area, velocity, velocity in anterior-posterior direction, acceleration and jerk in the medio-lateral direction (*p*<0.05, Fig. 1). In the semitandem stance with eyes closed condition, the real trial showed an increase of velocity in the medio-lateral direction and a decrease of global and medio-lateral jerkiness, compared to sham intervention (Table 1).

**Table 1:** Clinical and functional mobility parameters and static balance results in tandem and semitandem stances with eyes closed, before and after sham vs real cerebellar rTMS intervention performed in 20 patients with PSP

Variable	Pre-SHAM	Post-SHAM	Pre-REAL	Post-REAL	*p*
Tandem eyes closed (*n*=14)					
30-s task completed, *n*	9	7	8	7	0.8
Time, s	21.1 + 12.2	19.7 + 13.7	20.9 + 11.8	22.5 + 11.2	*0.046*
Area, mm^2^	6.55 + 5.71	12.81 + 11.66	25.42 + 24.06	12.79 + 15.26	*0.007*
Velocity					
MV, mm/s	158.50 + 33.37	206.89 + 102.57	289.38 + 242.22	202.87 + 85.49	*0.044*
MV-AP, mm/s	20.04 + 10.14	27.76 + 18.67	35.97 + 23.34	24.42 + 13.72	*0.009*
MV-ML, mm/s	77.99 + 65.36	103.62 + 94.79	134.97 + 199.56	89.87 + 71.01	0.299
Acceleration					
ACC, mm/s^2^	27.01 + 12.16	36.45 + 20.24	48.80 + 29.57	34.90 + 19.13	*0.005*
ACC-AP, mm/s^2^	62.10 + 41.89	68.20 + 48.91	84.01 + 72.00	76.32 + 46.67	0.544
ACC-ML, mm/s^2^	0.97 + 0.80	1.05 + 0.80	1.94 + 2.84	2.98 + 5.49	0.575
Jerk					
Jerk, mm/s^3^	6.66 + 6.60	8.14 + 6.36	19.86 + 30.36	7.42 + 5.33	0.069
Jerk-AP, mm/s^3^	1.02 + 0.76	1.69 + 2.06	1.99 + 2.70	1.92 + 2.97	0.514
Jerk-ML, mm/s^3^	17.53 + 8.25	22.92 + 9.97	31.68 + 20.66	24.20 + 14.72	*0.040*
Frequency					
MF, Hz	1.34 + 0.36	1.25 + 0. 37	1.29 + 0.51	1.27 + 0.34	0.647
Semitandem eyes closed (*n*=16)					
30-s task completed, *n*	9	9	9	9	1
Time, s	26.7 + 7.0	25.57 + 9.3	26.5 + 12.5	28.5 + 3.9	*0.046*
Area, mm^2^	4.37 + 3.63	5.57 + 4.31	11.07 + 15.29	4.35 + 3.01	0.085
Velocity					
MV, mm/s	139.06 + 23.53	149.12 + 38.57	192.49 v 98.08	143.03 + 40.02	0.083
MV-AP, mm/s	16.76 + 5.28	17.72 + 10.04	22.38 + 12.96	17.26 + 7.34	0.167
MV-ML, mm/s	106.51 + 61.66	72.27 + 63.99	85.49 + 86.10	94.17 + 75.60	*0.040*
Acceleration					
ACC, mm/s^2^	22.23 + 7.89	25.28 + 10.88	30.68 + 19.53	22.19 + 8.42	0.065
ACC-AP, mm/s^2^	51.73 + 32.22	64.44 + 38.06	57.27 + 33.94	68.90 + 69.31	0.956
ACC-ML, mm/s^2^	0.98 + 0.75	1.31 + 1.71	1.03 + 0.57	1.70 + 1.00	0.590
Jerk					
Jerk, mm/s^3^	3.98 + 2.47	3.87 + 2.15	7.29 + 4.61	3.51 + 0.90	*0.021*
Jerk-AP, mm/s^3^	1.03 + 0.74	2.01 + 3.49	1.74 + 2.52	0.92 + 1.25	0.164
Jerk-ML, mm/s^3^	14.08 + 7.14	17.28 + 6.86	20.56 + 15.27	13.63 + 5.18	*0.042*
Frequency					
MF, Hz	1.26 + 0.33	1.23 + 0.37	137 + 0.35	1.32 + 0.33	0.845

*ACC* acceleration, *AP* anterior-posterior, *MF* mean frequency, *ML* medio-lateral, *mm* millimetre, *MV* mean velocity, *RMS* root mean square, *s* seconds

Results from the stance tasks with eyes open showed similar results, although less pronounced. Dynamic mobility assessment did not show differences in performance for real vs sham trial (Supplementary Table 1).

## Discussion

Postural instability and falls are still important unmet therapeutic targets in PSP. The present trial, using a double-blind sham-controlled crossover design, suggests a beneficial effect of a single-session of cerebellar rTMS stimulation on measures of postural instability in PSP patients. Our results fit with the observation of alterations of the cerebellum in PSP [2, 3, 5, 6], and with preliminary evidence coming from pilot studies.

The first published rTMS open-label trial in PSP using cerebellar theta burst stimulation in 10 patients showed an improvement of functional connectivity between cerebellum and motor cortex assessed by neurophysiological measures (i.e. CBI) and functional MRI [7]. However, the trial could not exclude a placebo effect due to the open-label design. Moreover, the authors could not demonstrate a relevant clinical effect. This aspect was recently addressed by a sham-controlled rTMS case study performed for 10 days in two PSP patients, showing an improvement in CBI and posturography in the real intervention, although not significant due probably to an unexpected placebo effect in one patient [8].

The present trial adds novel insights into this concept on multiple levels. First, it provides information about a reasonably large cohort of early stages PSP and uses a high-quality design. Second, it considered several assessment strategies, including novel mobile health technology, to assess even subtle but potentially clinically relevant parameters. At variance with previous studies applying multiple sessions, a single theta burst stimulation showed a surprisingly clear effect on accelerometer-derived measures of static balance, a feature that is regularly affected in PSP and leads to severe impairment in daily activities and quality of life. The real intervention showed an effect particularly in the medio-lateral direction of static balance (see, e.g. Table 1 and Supplementary Table 1). This is of interest, as MacKinnon and colleagues [13] and Mitchell and colleagues [14] found that medio-lateral parameters of static balance reflect predominantly axial and antero-posterior parameters predominantly distal compensatory movements. In our assessments, PSP patients were able to extend the time they could perform the tasks after real stimulation, making us optimistic for the clinical translation of rTMS stimulation. Third, the improvement in static balance, as observed with mobile health technology, was not reflected by any of the clinical and mobility performance test included in the assessment battery. This result highlights the need for inclusion of such technology in these types of trials, as conventional assessment methods may be too roughly scaled to detect relatively subtle changes. Fourth, the effects of rTMS on static balance parameters were particularly evident in the more challenging tasks. This aspect argues for the usefulness of challenging paradigms to be included in assessment panels of clinical trials together with neuronavigation and more complex measures (including unsupervised assessments [15]). The response of a single-session rTMS could also identify those patients who might benefit most of longer rTMS trials to be performed in the future.

In conclusion, this is the first study showing a relevant effect of a short cerebellar rTMS intervention on static balance in PSP patients, supporting the rationale for longer stimulation protocols.

## Supplementary Information

ESM 1 (DOCX 638 kb)
