# Supplementary material for: Cerebellar rTMS in PSP: a Double-Blind Sham-Controlled Study Using Mobile Health Technology
Source: Cerebellum. 2021 Feb 5;20(4):662–6. doi: 10.1007/s12311-021-01239-6 (PMC8360898; doi:10.1007/s12311-021-01239-6)
Supplement: Supplementary file 1 — (DOCX 638 kb) [file 12311_2021_1239_MOESM1_ESM.docx]

**Supplementary Material**

**Recruitment and subtype of PSP**

The study included twenty PSP patients, classified according to currnt criteria () as PSP-RS (n=16), PSP-P (n=3) and PSP-PAGF (n=1).

**Resting Motor Threshold**

The resting motor threshold was defined via visual inspection as the minimal stimulus intensity needed to produce motor evoked potentials (MEPs) with an amplitude of at least 50 μV in 5 out of 10 consecutive trails during complete muscle relaxation, which was controlled by visually checking the absence of EMG activity at high-gain amplification (Rossini et al. 2015).

**Static Balance Assessment**

All patients were supervised in reaching the starting position and arms flagged in self-chosen comfortable position in the middle of a standard room for testing using sport shoes. Each task started with a sound emitted by the tablet, which was only started after adequate instruction and having the OK of the participant that the task can be started. If the participant had to perform a compensatory step during the task, the task was stopped and the time between the sound and the compensatory step was noted. If the participant was not able to maintain the position and did a compensatory step before the task could be started, the task was considered as not being performed due to inability of the participant.

Tandem and semitandem positions were defined according to standard Short Physical Performance Battery protocol (Guralnik et al. 1994). For Semitandem position, the heel of one foot was placed to the side of the first toe of the other foot, with the participant choosing which foot to place forward.

**Postprocessing and analyses**

To ensure that only data obtained from the effective static balance phase was included in the analysis, all datasets were 1) counterchecked with the time noted down by the investigator for the specific task and 2) evaluated by visual inspection (CH) to make sure that no step signal as well as no large amplitudes that could not be explained by body sway was included in the final dataset.

Acceleration signals were processed and calculated as previously described ( Arnold et al. 2018, Mancini et al 2011, Maetzler et al. 2017); The following parameters were extracted: sway area, mean velocity, mean acceleration (root mean square (RMS)), jerk (indicating smoothness of compensatory movements ), and mean frequency (Hanakova et al 2015). Mean Velocity, RMS and jerk were calculated for both, anteroposterior (AP) and mediolateral (ML) directions.

**Bibliography**

Arnold C, Schulte C, Moscovich M, Sünkel U, Zaunbrecher L, Metzger F, et al. Cholinergic pathway SNPs and postural control in 477 older adults. Front Aging Neurosci. 2018;10:1–8.

Guralnik JM, Simonsick EM, Ferrucci L et al. A Short Physical Performance Battery Assessing Lower Extremity Function: Association With Self-Reported Disability and Prediction of Mortality and Nursing Home Admission. J Gerontol 1994; 49:M85–M94. doi: 10.1093/geronj/49.2.M85

Hanakova L, Socha V, Schlenker J, Cakrt O, Kutilek P. Assessment of postural instability in patients with a neurological disorder using a tri-axial accelerometer. Acta Polytech. 2015;55:229–36.

Mancini M, Horak FB, Zampieri C, Carlson-Kuhta P, Nutt JG, Chiari L. Trunk accelerometry reveals postural instability in untreated Parkinson’s disease. Park Relat Disord. 2011; 17(7):557-62.

Maetzler W, Mancini M, Liepelt-Scarfone I, Müller K, Becker C, van Lummel RC, et al. Impaired trunk stability in individuals at high risk for Parkinson’s disease. PLoS One. 2012;7: e32240.

Rossini PM, Burke D, Chen R, et al. Non-invasive electrical and magnetic stimulation of the brain, spinal cord, roots and peripheral nerves: Basic principles and procedures for routine clinical and research application. An updated report from an I.F.C.N. Committee. Clin Neurophysiol 2015;126:1071–107. doi:10.1016/j.clinph.2015.02.001.


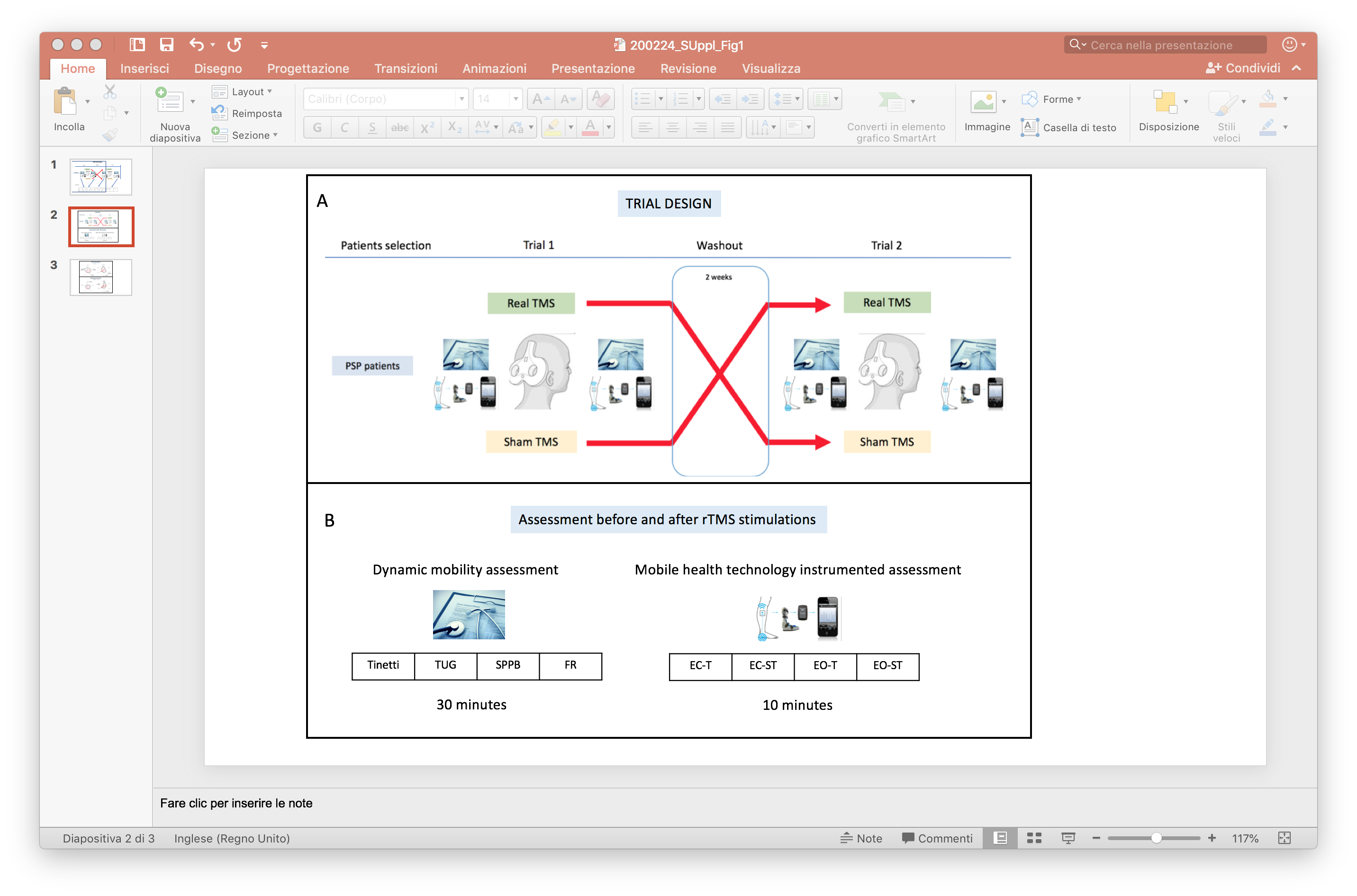


**Supplementary Figure 1 Study design and assessment**

**A** Crossover design of the rTMS study. After recruitment, 10 PSP patients underwent a real rTMS trial, and 10 were sham-treated. After a wash out period of at least two weeks, the second intervention was performed using a crossover design. **B** Assessments were performed before and after each rTMS session (real and sham), and included clinical scales and mobility assessment. Static balance tests were instrumented with mobile health technology (accelerometer device at the lower back, Rehagait®, Hasomed, Germany). EC-ST: semitandem static balance test performed with eyes closed; EC-T, tandem static balance test performed with eyes closed; EO-ST: semitandem static balance test performed with eyes open; EO-T: tandem static balance test performed with eyes open; FR, functional reach test; SPPB, Short Physical Performance Battery; TUG, Timed Up and Go test.

**Supplementary table 1** Results of static balance test in tandem and semitandem position and eyes open.

| **Variable** | **Pre-SHAM** | **Post-SHAM** | **Pre-REAL** | **Post-REAL** | **p** |
| --- | --- | --- | --- | --- | --- |
| ***Tandem stance eyes open (n=20)*** | | | | | |
| 30s task completed, n | 13 | 13 | 13 | 13 | 0.8 |
| TIME, s | 23.1 + 11.7 | 23.8 + 10.3 | 23.1 + 11.7 | 25.3 + 8.5 | 0.353 |
| AREA, mm^2^ | 6.12 + 8.62 | 6.55 + 8.56 | 11.58 + 16.94 | 7.14 + 10.50 | 0.423 |
| VELOCITY |  |  |  |  |  |
| MV, mm/s | 147.72 + 28.66 | 164.68 + 64.35 | 178.40 + 63.23 | 147.57 + 45.65 | **0.050** |
| MV-AP, mm/s | 16.93 + 10.37 | 18.54 + 10.76 | 21.83 + 11.96 | 20.81 + 21.68 | 0.728 |
| MV-ML, mm/s | 80.31 + 52.98 | 93.57 + 83.26 | 111.31 + 67.16 | 79.43 + 63.33 | 0.225 |
| ACCELERATION |  |  |  |  |  |
| ACC, mm/s^2^ | 23.93 + 12.62 | 24.33 + 13.83 | 32.23 + 22.34 | 26.62 + 22.34 | 0.527 |
| ACC-AP, mm/s^2^ | 71.09 + 37.19 | 42.13 + 20.37 | 73.64 + 45.80 | 65.19 + 57.77 | 0.273 |
| ACC-ML, mm/s^2^ | 1.13 + 1.14 | 0.83 + 0.92 | 0.94 + 1.00 | 1.05 + 0.73 | 0.358 |
| JERK |  |  |  |  |  |
| JERK, mm/s^3^ | 5.14 + 4.37 | 6.67 + 8.05 | 8.84 + 8.39 | 5.40 + 5.27 | 0.094 |
| JERK-AP, mm/s^3^ | 2.01 + 1.56 | 1.35 + 0.66 | 1.45 + 1.27 | 1.07 + 0.82 | 0.660 |
| JERK-ML, mm/s^3^ | 16.62 + 8.34 | 15.37 + 9.45 | 23.04 + 19.76 | 15.40 + 9.56 | 0.315 |
| FREQUENCY |  |  |  |  |  |
| MF, Hz | 1.36 + 0.36 | 1.46 + 0.47 | 1.34 + 0.45 | 1.41 + 0.53 | 0.813 |
| ***Semitandem stance eyes open (n=20)*** | | | | | |
| 30s task completed, n | 17 | 17 | 17 | 17 | 0.8 |
| TIME, s | 29.62 + 1.49 | 28.43 + 3.44 | 23.1 + 11.7 | 29.56 + 1.75 | **0.050** |
| AREA, mm^2^ | 3.63 + 3.03 | 5.16 + 8.64 | 8.48 + 15.62 | 3.53 + 1.88 | 0.156 |
| VELOCITY |  |  |  |  |  |
| MV, mm/s | 127.48 + 15.52 | 142.17 + 40.13 | 145.72 + 50.57 | 132.71 + 28.05 | 0.135 |
| MV-AP, mm/s | 15.01 + 5.12 | 14.47 + 6.51 | 21.40 + 26.96 | 14.84 + 4.03 | 0.385 |
| MV-ML, mm/s | 99.77 + 60.45 | 66.06 + 41.70 | 121.79 + 184.79 | 67.25 + 23.83 | 0.677 |
| ACCELERATION |  |  |  |  |  |
| ACC, mm/s^2^ | 19.78 + 6.94 | 22.20 + 14.00 | 27.20 + 28.16 | 19.82 + 5.55 | 0.203 |
| ACC-AP, mm/s^2^ | 60.20 + 43.89 | 74.13 + 70.48 | 48.25 + 35.51 | 74.18 + 53.35 | 0.588 |
| ACC-ML, mm/s^2^ | 0.93 + 0.83 | 1.79 + 0.81 | 1.39 + 1.11 | 0.73 + 0.78 | **0.002** |
| JERK |  |  |  |  |  |
| JERK, mm/s^3^ | 3.28 + 1.30 | 5.57 + 7.07 | 9.94 + 18.53 | 3.45 + 1.97 | 0.104 |
| JERK-AP, mm/s^3^ | 1.03 + 0.53 | 1.32 + 1.19 | 1.17 + 1.02 | 0.98 + 0.68 | 0.247 |
| JERK-ML, mm/s^3^ | 12.51 + 5.68 | 16.32 + 13.16 | 15.61 + 10.66 | 12.86 + 4.71 | 0.112 |
| FREQUENCY |  |  |  |  |  |
| MF, Hz | 1.29 + 0.36 | 1.39 + 0.44 | 1.361 + 0.44 | 1.31 + 0.42 | 0.178 |
| **Clinical assessment and motor tasks** | | | | | |
| Tinetti, total score | 16.9 + 4.5 | 17.0 + 4.2 | 16.8 + 4.5 | 17.5 + 4.3 | 0.071 |
| TUG, seconds | 20.8 + 10.5 | 19.9 + 9.57 | 22.3 + 9.57 | 21.6 + 7.41 | 0.882 |
| SPPB, total score | 5.90 + 2.50 | 5.95 + 2.48 | 5.85 v 2.41 | 5.90 + 2.35 | 0.894 |
| Functional reach, cm | 17.1 + 5.15 | 18.7 + 6.52 | 17.0 + 5.17 | 19.0 + 5.00 | 0.803 |

**Abbreviations:** ACC, acceleration; AP, anterior-posterior; cm, centimetre; FR, functional reach test; MF, mean frequency; ML, medio-lateral; mm, millimeter; MV, mean velocity; RMS, Root mean square, s, seconds; SPPB, Short Physical Performance Battery; TUG, Timed Up and Go test.
